# Evaluating Brewers’ Spent Grain Protein Isolate Postprandial Amino Acid Uptake Kinetics: A Randomized, Cross-Over, Double-Blind Controlled Study

**DOI:** 10.3390/nu15143196

**Published:** 2023-07-19

**Authors:** Meeke Ummels, Lonneke JanssenDuijghuijsen, Jurriaan J. Mes, Claire van der Aa, Ron Wehrens, Diederik Esser

**Affiliations:** 1 Wageningen Food & Biobased Research, Wageningen University & Research, 6708 WG Wageningen, The Netherlands; meeke.ummels@wur.nl (M.U.); lonneke.janssenduijghuijsen@wur.nl (L.J.); jurriaan.mes@wur.nl (J.J.M.); 2 EverGrain International, 3000 Leuven, Belgium; claire.vanderaa@everingredients.com; 3 Biometris, Wageningen University & Research, 6708 PB Wageningen, The Netherlands; ron.wehrens@wur.nl

**Keywords:** barley, brewer’s spent grain (BSG), EverPro, proteins, amino acid uptake, protein quality, upcycling

## Abstract

Valorization and utilization of brewers’ spent grain (BSG) are of great interest in terms of reducing food waste and promoting more sustainable food systems. In this study, we aimed to evaluate the nutritional value of upcycled barley/rice proteins (BRP) extracted from BSG and compare this with pea proteins (PP). A randomized, cross-over, double-blind controlled trial was conducted with twelve participants (age: 24 ± 2.8 years, BMI: 23.3 ± 3.0 kg/m^2^). During three separate visits with a one-week washout period between visits, participants received 20 g BRP, PP, or the benchmark protein whey (WP). Blood-free amino acids (AA) were measured to determine postprandial AA uptake kinetics. The estimated total AA (TAA) uptake of BRP was 69% when compared to WP and 87% when compared to PP. The time to reach the maximum values was similar between the three protein sources. When comparing individual essential AA responses between BRP and PP, we observed higher responses in methionine and tryptophane and lower responses in lysine, histidine, and isoleucine for BRP compared to PP. This study demonstrates that BRP exhibits comparable postprandial TAA uptake profiles to PP. The findings highlight the complementarity of BRP and PP, which may offer the potential for blending approaches to optimize protein quality for overall health.

## 1. Introduction

Nowadays, animal-derived proteins account for about 45% of human total protein consumption in developed countries [1]. The growing standards of living in developing nations and the increase in population are the primary factors driving even greater demand for animal-derived protein [2,3,4]. However, the current production of animal-derived protein, even after intensification, cannot keep up with this high demand [4] and is putting pressure on the planet’s resources. Therefore, there is a need to transition towards diets that include fewer animal-derived proteins and more sustainable alternatives. This transition will offer benefits for the environment, animal welfare, and human health [3].

Regarding sustainability, reducing food loss and waste is of significant importance. Currently, around one-third of the global food production intended for human consumption goes uneaten [5]. Food loss, as defined by the Food and Agriculture Organization (FAO) of the United Nations, refers to products meant for human consumption that are either not consumed by people or have experienced diminished quality in terms of their nutritional value, economic value, or food safety [6]. A considerable amount of the food produced is lost before it even reaches the end users, occurring during agricultural production (21–33%) or during product manufacturing, storage, distribution, and processing (21–25%) [5,7].

In the brewing industry, the most commonly used grain for the production of beer-type beverages, often in combination with adjunct grains such as rice and corn, is barley [8]. The annual global production of brewing waste in this industry is 39 million tons [9]. With the rapid development of the brewing industry, these numbers are likely to increase even further. Brewer’s spent grain (BSG) constitutes approximately 85% of the total brewing waste [10]. Until now, BSG has primarily been utilized as animal feed or, to a lesser extent, discarded directly, leading to resource waste and environmental pollution [11]. Given that BSG is the most voluminous by-product of the brewing industry, the valorization and utilization of spent grain protein is of great interest in terms of sustainability [8].

Upcycling side streams that are suitable for human consumption presents an excellent opportunity to meet the increasing demand for sustainable protein sources while simultaneously reducing food loss. Utilizing BSG would allow producers to repurpose protein-rich and nutrient-rich barley [12]. In addition to technical feasibility, assessing the protein quality is important before implementation in potential upcycling processes. Consequently, there is a growing interest in evaluating the protein quality of emerging sustainable protein sources. The hydrolysis of dietary protein into smaller compounds (tripeptides, dipeptides, or single amino acids (AA)) by proteases and peptidases in the small intestinal lumen, followed by their absorption into the bloodstream, is crucial for their nutritional value. Numerous plant proteins have been previously tested, with some commonly used in products as substitutes for dairy and/or meat products. Notably, a significant number of these market-available products incorporate pea protein isolates [13].

BSG is rich in cellulose and non-cellulosic polysaccharides, lignin, and proteins [14], making it a valuable resource for human nutrition and health. On average, BSG consists of 20% protein in terms of dry matter [8], with approximately 30% being essential amino acids (EAA) [15]. The BSG protein fraction consists mainly of hordeins (30–50%) and a mix of albumins, globulins, and glutelin [8]. In general, cereal products are low in the EAA lysine. Within this category of cereals, however, compared to grains like wheat and rice, BSG has a relatively high content of lysine [9,16]. Additionally, BSG is rich in bioactive compounds and micronutrients [17,18]. Due to its high nutritional value, BSG can be applied in human food products for fortification purposes [9]. Upcycled barley/rice protein (BRP) is enzymatically extracted from the BSG and purified via downstream processes, including filtration steps. The resulting BRP isolate can be applied in a range of products to enhance their protein content (e.g., plant-based milk, coffees, or smoothies) [19]. A significant degree of protein degradation occurs during the brewing process, resulting in a BSG protein isolate with a substantial high density of small peptides of all molecular weights [19]. This potentially aids in the digestibility of upcycled barley protein obtained from the beer brewing industry. The objective of the current study was to evaluate the postprandial AA uptake kinetics of BRP in a healthy adult population, aiming to assess its protein quality for human consumption. Furthermore, the study aimed to compare these outcomes with those of pea protein isolate (PP), which is frequently used in the plant protein market, as well as with the widely consumed benchmark, whey protein isolate (WP). As protein intake can also affect insulin responses, this study also investigated postprandial plasma glucose and insulin responses.

## 2. Materials and Methods

### 2.1. Ethical Consideration

All participants provided informed consent for participation in the study. This study was conducted in accordance with the Declaration of Helsinki, and the protocol was approved by the Ethics Committee of Radboud University Medical Center (Nijmegen, The Netherlands; NL80654.091.22). This study was registered at clinicaltrials.gov (NCT05426122). Study execution was between June and July 2022 at Wageningen University and Research, Wageningen, The Netherlands.

### 2.2. Study Participants

Participants were recruited via advertisements on social media and in newspapers, and via a database of Wageningen University and Research, between May and June 2022. Inclusion criteria were body mass index (BMI) between 18.5–30 kg/m^2^, age between 18–40 years, and having veins suitable for blood sampling via a peripheral venous catheter (judged by the medical research staff). The main exclusion criteria included possessing any metabolic, gastrointestinal, inflammatory, or chronic disease, using medication or supplements that could influence the study outcome, having anemia (Hb values < 7.5 mmol/L for women and <8.5 mmol/L for men), following slimming or medical prescribed diets, having weight loss or weight gain of >5 kg in the month before screening, using protein supplements, having food allergies and/or intolerances, being pregnant, lactating, or wishing to become pregnant in the period of the study and currently smoking. Eligibility was assessed using a screening visit, during which a questionnaire was administered to check medical history, veins were judged for repetitive blood collections, and BMI was measured.

### 2.3. Study Design and Procedures

**Figure 1:**
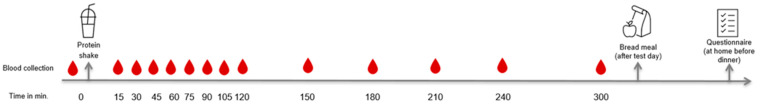
Overview of a test day.

This study had a randomized, cross-over, double-blind, controlled design. All participants visited the research unit on three occasions, with a washout period of one week between the test days. They were instructed not to drink alcohol or to perform heavy exercise the day prior to each test day. They consumed a standardized meal the evening before each test day and came to the research unit after an overnight fast. During each test day, participants received, in randomized order, one of the following three protein shakes: Barley/Rice (ratio on average 70/30) protein isolate (EverPro, EverGrain^®^ by AB InBev, St. Louis, MO, USA), pea protein isolate (NUTRALYS^®^ S85 XF, Roquette, Lestrem, France) or whey protein concentrate (Impact Whey Protein, MyProtein, Northwich, UK). BRP, PP, and WP were dissolved in 400 mL of cold water. The protein shakes were freshly prepared on the morning of each test day. Participants consumed the protein shakes at room temperature within a timeframe of 10 min. The amount/volume of the protein shakes was the same for each participant, with each shake containing 20 g of protein. Blood was collected via a catheter before (T = 0) and up to five hours after protein shake consumption (T = 30, 45, 60, 90, 120, 150, 180, 240, and 300 min). Well-being, health complaints, and adverse effects were monitored during the test days. At the end of each test day, participants received a bread-based meal at the research location. After each test day participants were asked to fill in an online questionnaire on gastrointestinal complaints before dinner at home. A schematic overview of a test day is shown in Figure 1.

### 2.4. Intervention Products

The purity of the protein powders ranged from 80% (PP) to 82% (WP) up to 85% (BRP), which equaled 25 g of PP, 24 g of WP, and 23 g of BRP added to result in 20 g protein in each shake. Maltodextrin was added to the PP shake (1.6 g) and the WP shake (0.6 g) to match the higher carbohydrate content of the BRP shake. Table 1 shows the energy and macronutrient content of the three intervention shakes (per consumption).

The AA profiles of the three protein powders were analyzed at Eurofins Scientific (Eurofins Analyses Nutritionnelles, Nantes, France) and are displayed in Table 2. The AA measured included alanine (Ala), arginine (Arg), the combination of Asparagine (Asp) and aspartic acid (Asn), cysteine (Cys), the combination of glutamine (Gln), glutamic acid (Glu), glycine (Gly), histidine (His), isoleucine (Ile), leucine (Leu), lysine (Lys), methionine (Met), phenylalanine (Phe), proline (Pro), serine (Ser), threonine (Thr), tryptophane (Trp), tyrosine (Tyr), and valine (Val).

### 2.5. Study Measures

#### 2.5.1. Blood Amino Acids

Nineteen free AA were determined in all collected blood samples. Blood samples were immediately processed after each collection, and plasma samples were stored at −80 °C until analyses. The blood samples were analyzed as described by Mes et al. [20] and based on the Waters AccQ-Tag ultra-derivation kit and HPLC method (Waters™ Chromatography B.V., Etten-Leur, The Netherlands) for AA analyses.

#### 2.5.2. Blood Glucose and Insulin

Plasma insulin and glucose levels were determined in all blood samples collected. Blood samples were immediately processed, and plasma samples were stored at −80 °C until further analyses at the Central Diagnostic Laboratory (CDL) (UMC Utrecht, Utrecht, The Netherlands).

#### 2.5.3. Gastrointestinal Symptoms 

After each test day, participants were asked to fill in an online questionnaire (Castor EDC, Amsterdam, The Netherlands) regarding any gastrointestinal symptoms.

### 2.6. Sample Size and Randomization 

Sample size was calculated based on the peak value and the total amount of free AA assessed in postprandial blood samples. Concerning the peak value, a difference of 100 μg/mL was regarded as interesting (=δ), with an individual difference in peak values of 100 μg/mL (=SD) [21,22]. A two-sided significance level of 5% and a power of 90% resulted in an estimated sample size of 11 participants. To account for potential drop-outs, the aim was to include 12 participants in this study. The treatment order was randomized by using blocked randomization (6 different treatment orders (3!); [ABC, ACB, BCA, BAC, CAB, CBA]). The variables of sex and age were stratified among the groups.

### 2.7. Statistical Analyses

Statistical analyses were performed using R statistics (R Foundation for Statistical Computing, Vienna, Austria) and IBM SPSS Statistics (Version 25.0. Armonk, NY, USA). The concentration of 19 individual free AA was quantified, and analysis was performed on the individual AA, total AA (TAA), and a total of nine essential AA (TEAA; Val, Trp, Thr, Phe, Met, Lys, Leu, Ile, and His). The time curves for postprandial AA levels in the blood of the twelve participants were fitted using the R-package ‘aaresponse’ specifically designed for analyzing AA response curves in cross-over intervention studies as previously described [20]. In cases where no peak was observed, data for peak height and iAUC were imputed, i.e., estimated from the raw data without curve fitting. This leads to unbiased estimates since non-responses are also taken into account, as well as more narrow confidence intervals. Corresponding estimates for time-2-max cannot be obtained because, in the absence of a peak, the peak position is undefined. A linear mixed model was used to compare the incremental area under the curve (iAUC), maximum peak height, and time to reach this maximum peak value (time-2-max) for the three protein powders, using BRP as a reference to compare the response of BRP to another often used plant protein source (pea), but also to compare the results against the commonly used and high-quality animal protein source whey (as a benchmark). This model comprised fixed effects for protein intervention and test week and a random effect for participants. The Kenward–Roger F test was used to assess the significance of the protein intervention effect. The differences between the 19 individual AA were explored by making use of descriptive analyses (mean differences + SD).

Glucose and insulin data were analyzed using the same mixed-model approach as used in the analysis of the AA. Differences in gastrointestinal symptoms between the three protein sources were analyzed using one-way ANOVA (SPSS Statistics). In all cases, a *p*-value of 0.05 was used as the threshold of statistical significance.

## 3. Results

### 3.1. Participants, Baseline Characteristics and Compliance

**Figure 2:**
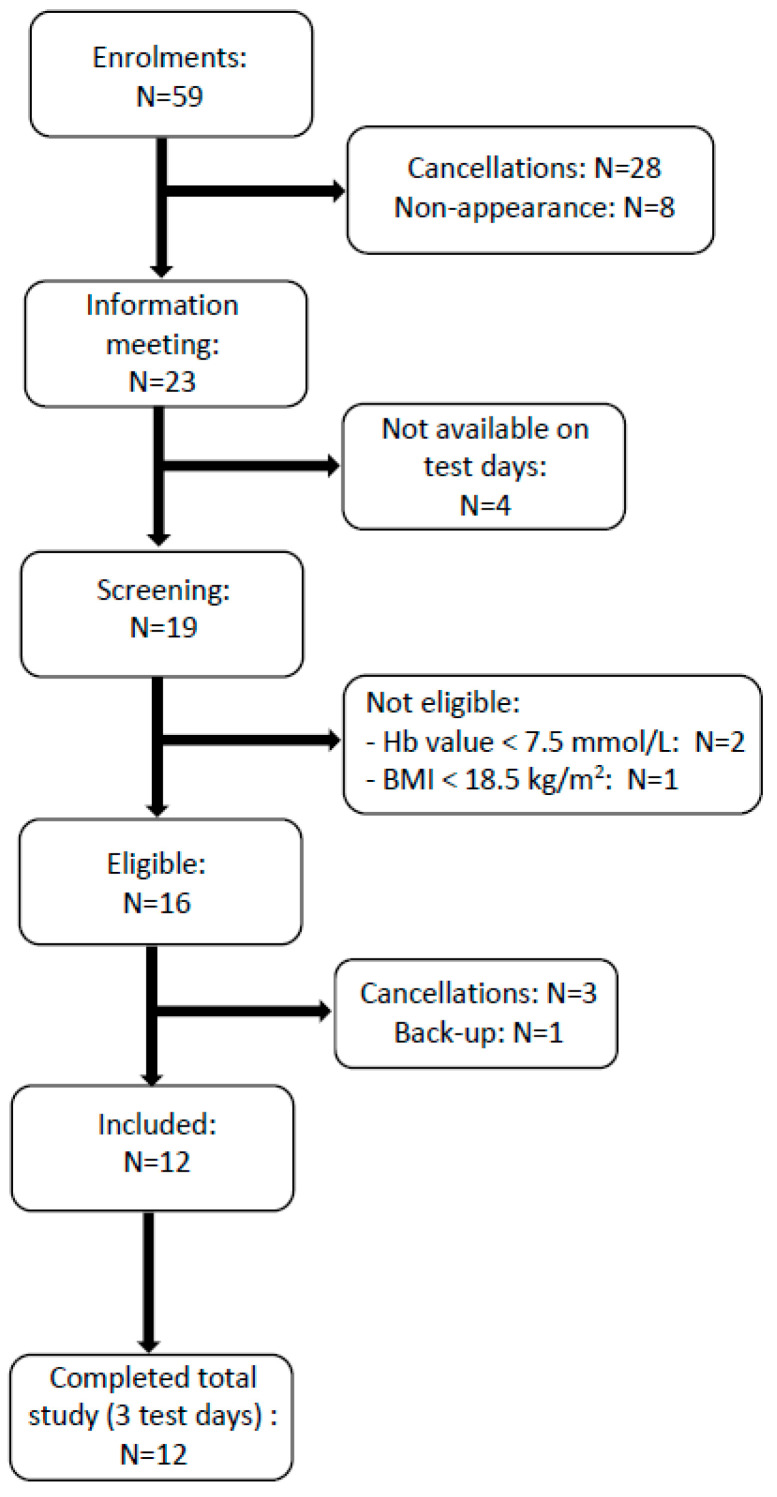
Flowchart diagram of study participant selection and inclusion.

Figure 2 shows a flowchart diagram of the participant selection via a screening procedure (as described in Section 2.2). All 12 participants (6 males and 6 females) completed the study. Their mean age was 24 ± 2.8 years (range: 20–30 years), and their mean BMI was 23.3 ± 3.0 kg/m^2^ (range: 20–28 kg/m^2^). 

### 3.2. Postprandial Amino Acid Responses

**Figure 3:**
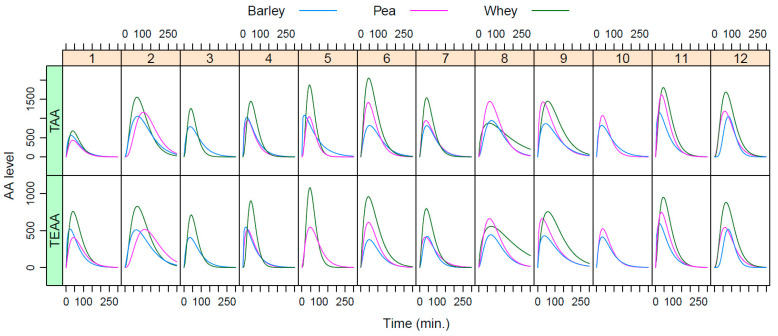
Postprandial AA levels per protein shake fitted per individual. For three individuals (3, 5, and 10), not all individual curves could be fitted based on absolute values (raw data, see Figure A1).

The individually plotted postprandial TAA and TEAA curves, as shown in Figure 3 illustrate interindividual differences in postprandial responses per product. Most individuals show higher peaks for TAA as well as TEAA after the WP benchmark shake consumption compared to the plant protein shakes consumption. The curves for BRP are in general similar, or lower than those for PP. There are large differences between individuals in maximum peak heights observed. From these fitted data, the iAUC, peak height, and time-2-max were calculated. 

Table 3 shows the iAUC, peak height, and time-2-max of the postprandial TAA and TEAA concentrations in blood after BRP, PP, and WP consumption. TAA and TEAA iAUC and peak height were significantly different in a three-way comparison between the three protein shakes. The uptake of TAA for BRP was 69% and 87% compared to the total AA uptake of respectively WP and PP. For the TEAA, the uptake for BRP was 58% and 82% compared to the TEAA uptake of, respectively, WP and PP. Time-2-max values for TAA and TEAA were all in the same range and not significantly different between the three protein shakes. 

**Figure 4:**
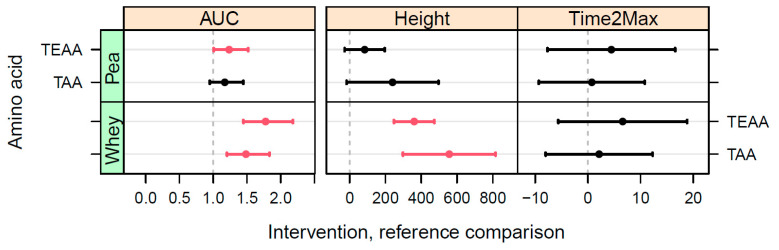
Confidence intervals for comparisons in iAUC, peak height, and time-2-max of TAA and TEAA between the PP and WP interventions on the one hand and the reference BRP on the other. For iAUC, the comparison is the ratio between the iAUC values (dimensionless); for the peak height and time-2-max, the comparisons are given by the absolute differences (µM or min, respectively). Red bars indicate a statistically significant difference (*p* < 0.05).

Statistical comparisons of the iAUC, peak height, and time-2-max between BRP, as the reference, and the other two interventions were made by using the confidence intervals from the fitted data. These confidence intervals, as shown in Figure 4, indicate that BRP had a significantly lower iAUC and peak height for both TAA and TEAA when compared to WP. When comparing BRP with PP, only the confidence interval of the TEAA was significantly lower for BRP than PP, no significant difference was found for TAA, both for iAUC and peak height. 

**Figure 5:**
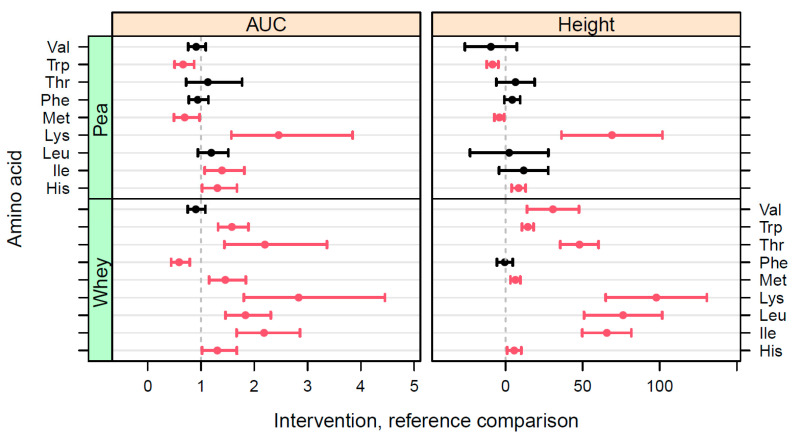
Confidence intervals for comparisons in iAUC and a peak height of individual EAA between the PP and WP interventions on the one hand and BRP as a reference on the other. For iAUC, the comparison is the ratio between the iAUC values (dimensionless); for the peak height, the comparison is given by the absolute differences (µM). Red bars indicate a statistically significant difference (*p* < 0.05), black bars indicate no statistically significant difference (*p* > 0.05).

**Figure A2:**
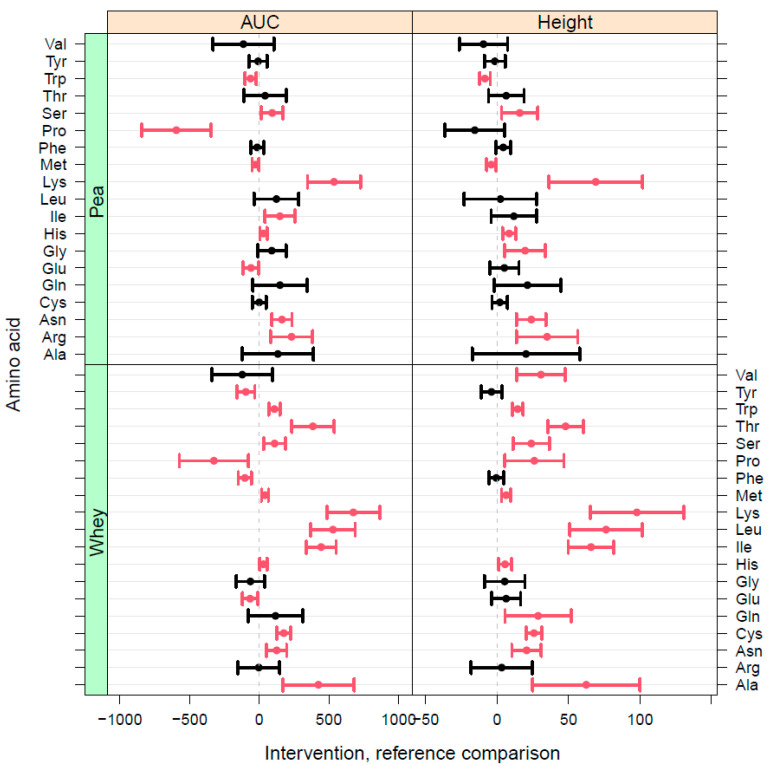
Confidence intervals for all comparisons of individual amino acids, after imputation compared to BRP. In cases where no peak was observed, data for the incremental area under the curve (iAUC) peak heights were imputed from the raw data without curve fitting. For the iAUC, the comparison is the ratio between the iAUC values of PP and WP intervention and the BRP intervention; for the peak height and time-2-max the comparison is given by the absolute differences (µM or min). Red bars indicate significant differences (*p* < 0.05) compared to the BRP intervention, black bars indicate no statistically significant difference (*p* > 0.05).

Curve fits were also performed for the individual AA. Outcomes of the iAUC, peak height, and time-2-max for each individual AA for each of the protein shakes are listed in supplemental Table A1, Table A2 and Table A3. Figure 5 shows the confidence intervals of the iAUC and peak height of the EAA for WP and PP compared to BRP after the imputation of cases where no peak was detected. Confidence intervals for the non-essential individual AA are shown in supplemental Figure A2. Seven out of the nine EAA are significantly higher in iAUC and peak height for WP compared to BRP. Phe, however, shows a higher uptake for BRP compared to the WP benchmark, while no significant difference is shown for Val. For all except Phe, the peak height was significantly higher for the WP benchmark than for BRP. When comparing the two plant protein isolates, the iAUC and peak height of Trp and Met were significantly higher for BRP, while the iAUC and peak height for Lys and His, as well as the iAUC of Ile were significantly higher for PP. No significant difference in the iAUC and peak height was observed for Val, Thr, Phe, and Leu between the two plant protein isolates.

**Figure 6:**
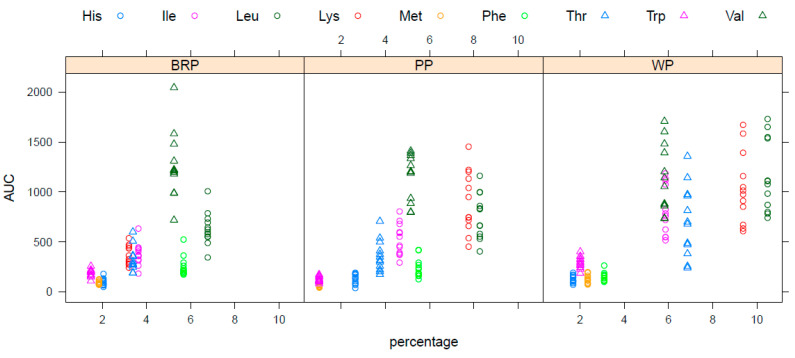
Comparison of the relative abundance of EAA in the protein (expressed in %) product against the iAUC response for each individual in blood after consumption of the protein source.

Figure 6 shows the iAUC of the AA taken up in the bloodstream expressed per AA content % within the respective protein shake. In general, the uptake of an AA into the bloodstream was predominantly reliant on the actual presence of that specific AA within the product. Nonetheless, certain variability between AA and substantial inter-individual variations were observed.

### 3.3. Glucose and Insulin Responses

**Figure 7:**
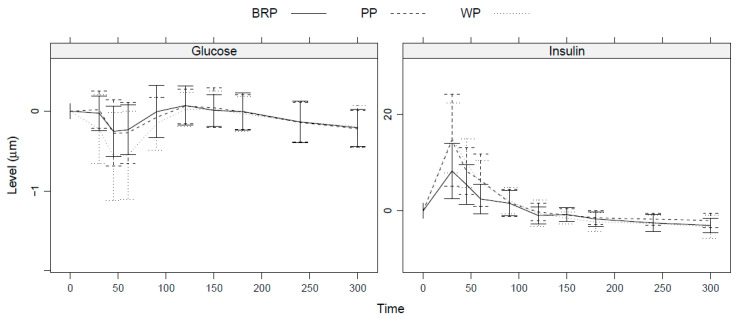
Postprandial glucose and insulin responses after BRP, PP, or WP shake consumption. Data show averages and SD across participants at each time point.

The postprandial insulin peak is lower for BRP than for WP and PP, see Figure 7. In contrast, the postprandial drop in glucose for BRP is similar to the drop seen for PP, while WP shows a larger drop in postprandial glucose levels. However, differences in iAUC and peak height were insignificant at the 5% level for both glucose and insulin.

### 3.4. Gastrointestinal Symptoms 

At the end of each test day, participants completed a short questionnaire on possible gastrointestinal symptoms. The mean outcomes are listed in Table 4. No significant differences in self-reported gastrointestinal symptoms and overall health were observed between the three protein shakes.

## 4. Discussion

We evaluated, in a double-blind, cross-over intervention trial, the postprandial AA uptake of protein from BRP and compared this response to PP and the benchmark protein WP. Differences in postprandial plasma TAA kinetics were observed between BRP, PP, and WP, with WP giving the highest response, followed by the two other protein sources. When looking at postprandial plasma TEAA confidence intervals, the response after BRP shake consumption was slightly lower when compared to PP. The time to reach the maximum values was similar between the three protein sources. Seven out of nine EAA showed higher postprandial responses after WP shake consumption when compared to BRP. BRP shake consumption did result in a higher uptake of the Phe when compared to WP. When comparing individual EAA responses between BRP and PP, we observed higher responses in methionine (Met) and tryptophane (TRP) and lower responses in lysine (Lys), histidine (His), and isoleucine (Ile) after BRP shake consumption when compared to PP. 

The estimated TAA uptake of BRP, as determined by comparing the calculated iAUC, is 69% compared to the benchmark WP. The EAA uptake of BRP is somewhat lower, with an estimated relative uptake of 58% compared to WP. Plant proteins exhibit reduced digestibility due to the presence of indigestible fractions within their sequence as well as the presence of anti-nutritional factors [23]. Hence, a higher uptake of WP was expected. We included a whey intervention as a benchmark since this protein is known for its optimal absorption and well-balanced EAA composition [24,25]. Since several other studies also include a whey intervention arm in their design, it allows us to calculate a relative uptake and estimate and compare effect sizes across studies that used a similar approach.

Previously, we conducted a comparable study on postprandial AA uptake investigating Lemna protein concentrate. In that publication, we also reported an overview of the available literature data on iAUC values of EAA uptake from other protein sources in a human cross-over design with whey protein as the benchmark [20]. From that overview, it is apparent that in today’s literature, postprandial AA uptake studies in humans comparing whey with plant proteins and or protein blends are still relatively scarce. A human postprandial study described by Lui et al. [26] reported a relative AA uptake of pea protein compared with whey protein that was somewhat lower, around 70%, than the relative TAA uptake we found in the current study, around 80%. The iAUC results for BRP fall within the range of those previously reported for casein, with studies reporting a relative uptake of TEAA ranging from 49% to 60% compared to whey [27,28,29,30]. Interestingly, it appears that BRP exhibits a higher estimated range of EAA uptake compared to other cereal proteins reported in the literature, which might be explained only partially by the rice component [16]. Gorissen et al. [28] evaluated postprandial plasma AA concentrations of wheat protein hydrolysate and compared them to casein and whey and reported an estimated TEAA uptake of 45% for wheat protein hydrolysate compared to whey. The brewing process, in combination with the processing methods applied to isolate BRP, may potentially impact its protein bioavailability [19]. It is, however, important to exercise caution when interpreting these comparisons, as direct comparisons between wheat, casein, and barley/rice were not made in our study. Furthermore, certain aspects, such as protein quality, processing steps, purity, or amount of protein ingested, could also differ between studies.

Statistically significant differences were not observed when examining the iAUC of the TAA for the two plant proteins investigated in this study. We did, however, observe a slightly greater overall response in TEAA following the consumption of the PP shake compared to the BRP shake. Even though the iAUC of TEAA for BRP was approximately 82% in comparison to PP, certain individual EAA clearly exhibited higher responses for BRP. Furthermore, no significant differences in the peak heights of TAA and TEAA were observed between BRP and PP. BRP has been shown to have substantially higher solubility in comparison to pea protein, with on average 102% (expressed as total protein percentage remaining in a 1% solution) and 22%, respectively, making BRP nearly five times more soluble than PP [19]. Due to its high solubility, it would be possible to administer higher BRP quantities in similar product applications, allowing to compensate for potential lower uptake. 

Next to the lower overall uptake rates of plant proteins, the absorption speed of plant proteins may also be delayed. For instance, higher time-2-max values of the TAA and TEAA responses were observed in a previous study with corn protein isolate (manuscript in preparation) and Lemna protein concentrate [20] when compared to whey as a benchmark protein source. Here, we demonstrate that the speed of BRP uptake, determined by the time needed to reach the maximal postprandial blood AA levels, was comparable to and in a similar range of PP and even in the same range of WP. Whey is considered a fast-acting protein, and its absorption rate has been estimated at ~10 g per hour [31]. These results indicate that BRP also has a relatively fast absorption rate.

This study provides valuable insights into the potential absorption rates of individual EAA. When a particular EAA is limited in availability in the diet, the synthesis of protein is hampered [32]. Therefore, estimations regarding the proportion of EAA taken up into the bloodstream may provide us with valuable information. Still, these outcomes on individual AA responses should be interpreted with caution since we did not correct for multiple testing. The effect sizes obtained in this study, however, provide valuable insights for future studies. For example, our findings showed substantial inter-individual variation in the percentage of individual EAA taken up into the blood after consumption of the specific protein sources. This implies that not only the rate-limiting EAA may differ between protein sources, but it may also vary between individuals. 

Since whey is considered a protein source with an optimal EAA composition and easy to digest [24], it is not surprising that 7 out of 9 EAA had higher postprandial responses after WP shake consumption compared to BRP. However, BRP shake consumption resulted in a higher postprandial uptake of Phe, an EAA which serves as a precursor for the synthesis of the catecholamines (dopamine, norepinephrine, and epinephrine). The synthesis and release rates of catecholamines are directly influenced by the availability of their precursor from blood. Therefore, Phe demonstrates potential for brain function, manifested through various effects such as analgesic and antidepressant properties [33]. Furthermore, since this EAA cannot be oxidized by the muscles, its uptake and incorporation into muscle are assumed to serve as accurate indicators of muscle protein synthesis [34]. We consider the finding on higher postprandial levels of Phe a potential interesting lead to study further. 

Uptake scores of individual EAA of BRP, estimated by comparing the iAUC, were in a higher (Met, Trp), similar (Val, Thr, Phe, Leu), or lower (Lys, His, Ile) range compared to PP. The higher postprandial uptake levels of Met and lower levels of Lys after consumption of the BRP shake were expected. This is because the AA composition of pea, in general, is characterized by a limited content of Met [35], whereas barley and other cereals such as wheat and rice, as mentioned earlier, are known to be low in Lys [16,36]. This makes them interesting complementary protein sources [16,32]. Previously conducted research indicates that Met serves as an important cellular antioxidant, stabilizes the structure of proteins, participates in the sequence-independent recognition of protein surfaces, and can act as a regulatory switch through reversible oxidation and reduction [37]. Higher levels of tryptophan (Trp) may also be beneficial as this AA and its metabolites, such as serotonin, play a key signaling role in the gut-brain axis, thereby modulating health effects [38,39]. 

Despite the significantly higher Glu content in BRP compared to PP and WP, as well as in relation to other AA, there was no significant difference observed in the postprandial iAUC of Glu among the three protein shakes. Additionally, the iAUC of Glu in relation to its presence in the product was generally low compared to other AA. One plausible explanation for the low concentration of Glu in the bloodstream is the high energy demand of the small intestine epithelium, particularly in the postprandial state. Consequently, Glu is either directly utilized or taken up from the bloodstream for extensive oxidation within the cells of the small intestine epithelium [40]. Moreover, Glu serves as a precursor for several other AA, including alanine, aspartate, ornithine, and proline, thus playing a role in interorgan metabolic processes [40]. 

In the current study, participants assessed their general health and reported their experiences of bloating, nausea, occurrence of belching, flatulence, diarrhea, and constipation using an online questionnaire after each test day. The occurrence of these gastrointestinal symptoms was minimal and did not significantly differ among the three protein shakes. This indicates that the consumption of 20 g BRP per day was well-tolerated. 

Despite having an equivalent carbohydrate composition, differences were observed in the postprandial insulin and glucose responses among the protein shakes. Consumption of the BRP shake, although not significant, led to a reduced peak in insulin levels, while the peak in glucose levels was comparable to that of the PP shake. This may be partly explained by differences in the type of carbohydrates, as WP and PP contained added maltodextrin to match the higher carbohydrate content of BRP. The breakdown of proteins into AA triggers an insulin response, and indeed, a rapid surge in insulin was observed shortly after protein ingestion. The purpose of this insulin surge is to facilitate the transportation of AA into the muscles, thereby stimulating muscle protein synthesis [41]. However, the specific nature of the insulin response may vary depending on the source of the protein, as emerging evidence suggests that substituting animal protein with plant protein yields better glycemic control [42]. The endocrine hormones GIP and GLP-1, whose secretion can be stimulated by certain dietary proteins, peptides, and AA, may potentially contribute to this process [43]. However, it is important to note that these hormones were not evaluated in the present study. 

The evaluation of protein digestibility and nutritional characteristics of emerging sustainable protein sources poses challenges, particularly when avoiding animal experiments. The gold standard methodologies for assessing digestibility, namely the protein digestibility-corrected AA score, PDCAAS, and the more recent digestible indispensable AA score, DIAAS [6], rely solely on animal experiments and lack the ability to capture variations in response among individuals. Although in vitro alternatives do exist, they are not sufficient as it has been proven difficult to accurately measure the bioavailable fraction. Accurately determining true AA digestibility in humans is methodologically complex and requires costly stable isotope techniques. Consequently, the reported AA digestibility in humans remains limited for a select range of proteins. In this study, we measured postprandial blood AA concentrations to estimate protein quality, recognizing that the appearance of free AA in the blood only partially reflects protein digestibility. The AA profile in the blood following a meal is influenced by factors such as splanchnic tissue metabolism, intestinal passage speed, protein uptake/breakdown, and endogenous synthesis. The latter not being relevant for the EAA, which are of more importance for human health. Comparing the postprandial response to a reference and measuring it over time allows us to minimize the impact of confounding factors. Postprandial AA kinetics, therefore, serve as a valuable characteristic for assessing the potential nutritional impact of a protein, particularly for novel protein sources. Compared to stable isotope techniques, this method is relatively straightforward to perform, making it an important tool for evaluating the protein quality of emerging protein sources. It is, however, important to note that this method does not provide a quantitative protein digestibility score. 

## 5. Conclusions

Plant protein concentrates and isolates are increasingly consumed and have become important ingredients in shaping future sustainable diets. Upcycling barley via its substantial side stream in the beer brewing industry would allow producers to repurpose protein-rich and nutrient-rich barley. In this study, we demonstrate that upcycled barley/rice protein from barley spent grain exhibits clear postprandial total AA uptake profiles. Although upcycled barley/rice protein had a slightly lower overall EAA uptake compared to that of pea protein, it showed higher uptake of methionine and tryptophane. Furthermore, the overall absorption speeds, as estimated by the time-2-max, were similar to both pea and whey proteins. The findings also highlight the complementarity of barley/rice and pea protein, which may offer future potential in blending approaches to optimize protein quality for overall health. Future research, including upcycled barley protein, should include examining the effects on health outcomes, such as skeletal muscle synthesis, as well as implications for product development, such as texture properties and sensory characteristics of different sources of BSG.

## Figures and Tables

**Table 1:** Nutritional content of BRP, PP, and WP shake per consumption, based on raw weight.

	BRP	PP	WP
	(23 g)	(25 g)	(24 g)
Purity	85%	80%	82%
Energy (kcal)	85.0	103.0	101.0
Carbohydrate, total (g)	1.6	1.6	1.6
Protein, total (g)	20.0	20.0	20.0
Fat, total (g)	0.2	2.3	1.8
Fiber, total (g)	0.5	0.3	-

**Table 2:** Amino acid content (gram per 100 g protein isolate).

Amino Acid ^a^ (g/100 g)	BRP	PP	WP
Ala	4.1 ± 0.6	3.2 ± 0.5	4.0 ± 0.6
Arg	4.1 ± 0.6	6.5 ± 0.9	1.9 ± 0.3
Asp	7.7 ± 1.1	8.9 ± 1.3	8.5 ± 1.2
Cys	1.5 ± 0.2	0.8 ± 0.1	1.9 ± 0.3
Glu	22 ± 3.1	13 ± 1.8	14 ± 1.9
Gly	3.8 ± 0.5	3.1 ± 0.4	1.4 ± 0.2
His *	1.9 ± 0.3	2.0 ± 0.3	1.4 ± 0.2
Ile *	3.3 ± 0.5	3.5 ± 0.5	4.7 ± 0.7
Leu *	6.1 ± 0.9	6.2 ± 0.9	8.4 ± 1.2
Lys *	2.9 ± 0.4	5.8 ± 0.8	7.5 ± 1.1
Met *	1.7 ± 0.2	0.8 ± 0.1	1.9 ± 0.3
Phe *	5.2 ± 0.7	4.1 ± 0.6	2.5 ± 0.4
Pro	10.1 ± 1.4	3.3 ± 0.5	4.6 ± 0.6
Ser	3.8 ± 0.5	3.9 ± 0.5	4.0 ± 0.6
Thr *	3.1 ± 0.4	2.8 ± 0.4	5.5 ± 0.8
Trp *	1.4 ± 0.1	0.8 ± 0.1	1.6 ± 0.2
Tyr	3.5 ± 0.5	2.9 ± 0.4	2.2 ± 0.3
Val *	4.8 ± 0.7	3.8 ± 0.5	4.7 ± 0.7

Mean ± standard deviations. * Essential amino acids. ^a^ Ala = alanine; Arg = arginine; Asp = asparagine + aspartic acid; Cys = cysteine; Glu = glutamine + glutamic acid; Gly = glycine; His = histidine; Ile = isoleucine; Leu = leucine; Lys = lysine; Met = methionine; Phe = phenylalanine; Pro = proline; Ser = serine; Thr = threonine; Trp = tryptophane; Tyr = tyrosine; Val = valine.

**Table 3:** iAUC, peak height, and time-2-max of TAA and TEAA after consumption of BRP, PP, and WP.

		BRP	PP	WP	*p*-Value
TAA	iAUC	6419 ± 1775	7371 ± 2930	9261 ± 3195	0.04
	Peak height	898 ± 160	1121 ± 335	1434 ± 418	<0.01
	Time-2-max	46 ± 22	47 ± 23	50 ± 11	0.86
TEAA	iAUC	3165 ± 755	3859 ± 1083	5435 ± 1576	<0.01
	Peak height	473 ± 67	545 ± 111	813 ± 159	<0.01
	Time-2-max	43 ± 21	48 ± 25	52 ± 13	0.60

Mean ± standard deviations calculated by using the automatically curated data. iAUC expressed in arbitrary units, peak height expressed as µM, and time-2-max in minutes.

**Table 4:** Mean gastrointestinal (GI) symptoms reported at the end of each test day (*n* = 12).

	BRP	PP *	WP	*p*-Value
General Health	80 ± 24	86 ± 12	83 ± 15	0.98
Bloated feeling	18 ± 28	15 ± 21	14 ± 14	0.32
Belching	10 ± 18	5 ± 6	9 ± 13	0.55
Flatulence	12 ± 18	7 ± 8	12 ± 20	0.36
Nauseous	15 ± 21	14 ± 19	19 ± 29	0.72
Diarrhea	5 ± 9	8 ± 12	14 ± 28	0.66
Constipation	12 ± 18	6 ± 10	6 ± 9	0.63

Mean ± standard deviation. All outcomes were reported on a visual analog scale (VAS) ranging from 0–100. * *n* = 11.

**Figure A1:**
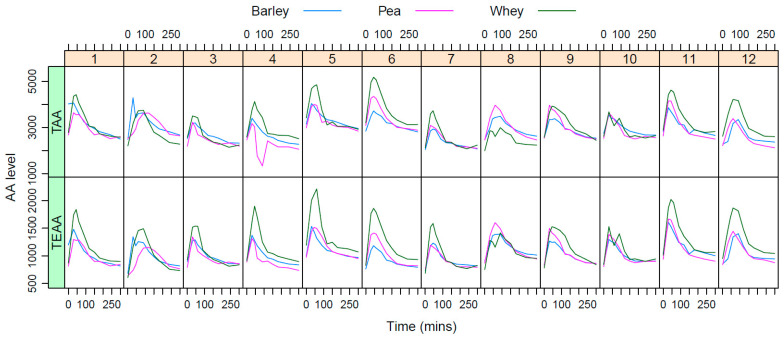
Raw postprandial AA levels per Barley/Rice protein (BRP), pea protein (PP), and whey protein (WP) protein per individual.

## Data Availability

Data are available upon substantiated request from the corresponding author. Data are not publicly available since subjects did not sign consent for this.

## Appendix B

**Table A1:** Incremental area under the curve (iAUC) of the individual amino acid concentrations after consumption of the Barley/Rice protein (BRP), pea protein (PP), and whey protein (WP) shake. iAUC expressed in arbitrary units.

Amino Acid	BRP	PP	WP	*p*-Value
Ala	733 ± 185 a	866 ± 234 a	1158 ± 395 b	<0.01
Arg	366 ± 95 a	597 ± 267 b	362 ± 131 a	<0.01
Asn	96 ± 30 a	259 ± 151 b	221 ± 85 b	<0.01
Asp	ND	ND	ND	-
Cys	69 ± 30 a	68 ± 30 a	245 ± 86 b	<0.01
Gln	515 ± 249	664 ± 382	632 ± 348	0.52
Glu	191 ± 93 a	131 ± 50 a	125 ± 77 a	0.08
Gly	288 ± 107 ab	377 ± 187 b	224 ± 109 a	0.04
His *	97 ± 36 a	126 ± 50 a	126 ± 36 a	0.14
Ile *	375 ± 111 a	523 ± 158 a	819 ± 248 b	<0.01
Leu *	633 ± 165 a	756 ± 227 a	1162 ± 361 b	<0.01
Lys *	368 ± 95 a	905 ± 309 b	1043 ± 354 b	<0.01
Met *	90 ± 18 a	62 ± 12 a	131 ± 46 b	<0.01
Phe *	253 ± 101 b	238 ± 97 b	150 ± 45 a	0.01
Pro	1065 ± 449 b	469 ± 156 a	739 ± 249 a	<0.01
Ser	239 ± 121	331 ± 207	348 ± 154	0.23
Thr *	321 ± 123 a	362 ± 157 a	705 ± 356 b	<0.01
Trp *	187 ± 39 b	124 ± 28 a	295 ± 61 c	<0.01
Tyr	364 ± 107 b	355 ± 89 ab	267 ± 65 a	0.02
Val *	1262 ± 334	1148 ± 232	1140 ± 340	0.56

Mean ± standard deviations calculated by using the automatically curated data. * Essential amino acids. Letters (a, b, and c) represent the Bonferroni post hoc test outcomes. Pairwise comparisons sharing the same letter are considered statistically similar, while different letters indicate statistically significant differences. ND = not detected.

## Appendix C

**Table A2:** Mean peak height of the individual amino acids after consumption of the Barley/Rice protein (BRP), pea protein (PP), and whey protein (WP) shake.

Amino Acid	BRP	PP	WP	*p*-Value
Ala	115 ± 21 a	135 ± 30 a	177 ± 58 b	<0.01
Arg	56 ± 17 a	91 ± 30 b	59 ± 25 a	<0.01
Asn	16 ± 4 a	40 ± 17 b	37 ± 12 b	<0.01
Asp	ND	ND	ND	-
Cys	10 ± 4 a	12 ± 4 a	36 ± 8 b	<0.01
Gln	86 ± 29	107 ± 41	115 ±53	0.23
Glu	20 ± 7	25 ± 15	26 ± 19	0.52
Gly	46 ± 9 a	66 ± 21 b	52 ± 16 ab	0.01
His *	19 ± 3 a	28 ± 5 b	25 ± 7 b	<0.01
Ile *	57 ± 10 a	69 ± 15 a	123 ± 27 b	<0.01
Leu *	103 ± 20 a	106 ± 25 a	180 ± 39 b	<0.01
Lys *	65 ± 11 a	134 ± 38 b	163 ± 46 b	<0.01
Met *	16 ± 2 b	12 ± 2 a	22 ± 5 c	<0.01
Phe *	29 ± 4 a	33 ± 8 a	28 ± 6 a	0.14
Pro	86 ± 23 ab	70 ± 23 a	112 ± 44 b	<0.01
Ser	35 ± 8 a	51 ± 19 ab	59 ± 18 b	<0.01
Thr *	44 ± 11 a	51 ± 17 a	92 ± 22 b	<0.01
Trp *	30 ± 4 b	21 ± 4 a	44 ± 7 c	<0.01
Tyr	44 ± 6 a	42 ± 8 a	40 ± 9 a	0.47
Val *	117 ± 14 a	108 ± 15 a	148 ± 27 b	<0.01

Mean (µM) ± standard deviations, calculated by using the automatically curated data. * Essential amino acids. Letters (a, b, and c) represent the Bonferroni post hoc test outcomes. Pairwise comparisons sharing the same letter are considered statistically similar, while different letters indicate statistically significant differences. ND = not detected.

**Table A3:** Mean Time-2-max of individual amino acids after consumption of the Barley/Rice protein (BRP), pea protein (PP), and whey protein (WP) shake.

Amino Acid	BRP	PP	WP	*p*-Value
Ala	52 ± 21 a	49 ± 22 a	49 ± 13 a	0.91
Arg	42 ± 19 a	43 ± 26 a	44 ± 12 a	0.97
Asn	46 ± 19 a	46 ± 26 a	46 ± 10 a	1.00
Asp	ND	ND	ND	-
Cys	65 ± 25 a	53 ± 26 a	62 ± 15 a	0.51
Gln	53 ± 21 a	51 ± 20 a	48 ± 13 a	0.84
Glu	81 ± 36 a	49 ± 22 a	53 ± 20 a	0.04
Gly	48 ± 21 a	45 ± 24 a	40 ± 7 a	0.59
His *	46 ± 17 a	46 ± 19 a	43 ± 11 a	0.88
Ile *	43 ± 18 a	51 ± 27 a	50 ± 14 a	0.66
Leu *	42 ± 19 a	49 ± 25 a	53 ± 12 a	0.46
Lys *	39 ± 18 a	45 ± 26 a	48 ± 11 a	0.60
Met *	41 ± 18 a	44 ± 15 a	47 ± 9 a	0.63
Phe *	48 ± 20 a	53 ± 22 a	41 ± 11 a	0.32
Pro	77 ± 37 b	46 ± 21 a	49 ± 13 a	0.01
Ser	45 ± 22 a	48 ± 26 a	44 ± 10 a	0.89
Thr *	46 ± 23 a	51 ± 27 a	56 ± 21 a	0.61
Trp *	50 ± 17 a	49 ± 15 a	55 ± 12 a	0.63
Tyr	48 ± 22 a	59 ± 24 a	50 ± 11 a	0.43
Val *	59 ± 27 a	66 ± 32 a	55 ± 16 a	0.65

Mean (µM) ± standard deviations, calculated by using the automatically curated data. * Essential amino acids. Letters (a and b) represent the Bonferroni post hoc test outcomes. Pairwise comparisons sharing the same letter are considered statistically similar, while different letters indicate statistically significant differences. ND = not detected.
